# Src Family Kinases (SFK) Mediate Angiotensin II-Induced Myosin Light Chain Phosphorylation and Hypertension

**DOI:** 10.1371/journal.pone.0127891

**Published:** 2015-05-26

**Authors:** Bo Qin, Junlan Zhou

**Affiliations:** 1 Weinberg College of Arts and Sciences, Northwestern University, Chicago, IL, United States of America; 2 Feinberg Cardiovascular Research Institute, Feinberg School of Medicine, Northwestern University, Chicago, IL, United States of America; Georgia Regents University, UNITED STATES

## Abstract

Angiotensin (Ang) II is the major bioactive peptide of the renin–angiotensin system (RAS); it contributes to the pathogenesis of hypertension by inducing vascular contraction and adverse remodeling, thus elevated peripheral resistance. Ang II also activates Src family kinases (SFK) in the vascular system, which has been implicated in cell proliferation and migration. However, the role of SFK in Ang II-induced hypertension is largely unknown. In this study, we found that administration of a SFK inhibitor SU6656 markedly lowered the level of systemic BP in Ang II-treated mice, which was associated with an attenuated phosphorylation of the smooth-muscle myosin-light-chain (MLC) in the mesenteric resistant arteries. In the cultured human coronary artery smooth muscle cells (SMCs), pretreatment with SU6656 blocked Ang II-induced MLC phosphorylation and contraction. These results for the first time demonstrate that SFK directly regulate vascular contractile machinery to influence BP. Thus our study provides an additional mechanistic link between Ang II and vasoconstriction via SFK-enhanced MLC phosphorylation in SMCs, and suggests that targeted inhibition of Src may provide a new therapeutic opportunity in the treatment of hypertension.

## Introduction

Hypertension affects approximately 78 million people in the United States, and is a major risk factor for coronary artery disease, congestive heart failure, stroke, end-stage renal disease, and peripheral vascular disease [1]. Current pharmacological therapy of essential hypertension primarily focuses on reducing vascular resistance by antagonizing vasoconstricting peptide hormones, such as Ang II and catecholamines, and calcium channels [2]. Vascular resistance is attributed primarily to the action of the contractile machinery, including actin and myosin filaments, in the vascular SMCs of the resistant vessels. Phosphorylation of the myosin’s regulatory light chain (MLC) subunits, particularly on Serine 19, is a key signaling event, which allows myosin to bind actin and use ATP to generate a force of contraction [3, 4]. The reaction is catalyzed by Ca2+/calmodulin-dependent MLC kinase and modulated by the activities of other kinases, such as Rho-associated kinase (ROCK), integrin-linked kinase (ILK), and zipper-interacting protein kinase (ZIPK) [3].

Ang II is the major bioactive peptide of the RAS and plays a critical role in cardiovascular homoeostasis and pathogenesis [5]. Previous studies have documented that Ang II induces vasoconstriction through multiple cellular signaling pathways. It activates AT1 receptor to couple G_q/11_ and G_i/o_ proteins, thus activates phospholipase C and increases the cytosolic Ca^2+^ concentrations, which in turn triggers the activation of Ca2+/calmodulin-dependent MLC kinase (thus actin-myosin motor activity), protein kinase C, MAPKs (ERK1/2, JNK, and p38 kinase), and tyrosine kinases including SFK [6–10].

Accumulating evidence suggests that SFK activation is one of the early events in Ang II-induced signal transduction, and that SFK play an important role in Ang II-induced vascular responses, such as cell proliferation via ERK1/2 activation [11], cell migration [12] and contraction [13]. However, how SFK contribute to arterial contractile response and whether SFK have a role in Ang II-induced hypertension are currently not known. Here we provide evidence that SFK are required for Ang II-induced MLC phosphorylation and hypertension; thus targeting SFK may have therapeutic implications for blood pressure (BP) disorders.

## Materials and Methods

### Animals and Ethics Statement

C57BL mice were purchased from JAX Lab. All breeding, maintenance, and experimental procedures were approved by the Institutional Animal Care and Use Committee of Northwestern University (animal study protocol# 2010–1957) and conducted at the University's Center for Comparative Medicine. Mice were maintained on a 12-hour/12-hour light/dark cycle with food and water provided ad libitum.

### Induction of Hypertension and BP Measurement

For induction of hypertension, 8-week-old male mice were administered Ang II (1.4 mg/kg/d, Sigma-Aldrich, St. Louis, MO) continuously for 14 days via a subcutaneous osmotic minipump (Alzet Model 100.2, DURECT Corporation), which was implanted at the right side back of mice with a minor surgical procedure in the isoflurane-anesthetized animals. A portion of mice also received i.p. injection of Src inhibitor SU6656 (8 mg/kg/d, Sigma-Aldrich, St. Louis, MO) during the last 2 days (day 13 and 14) of Ang II treatment. Arterial systolic, diastolic, and mean BP were measured by the standard noninvasive tail-cuff method (CODA System, Kent Scientific, Torrington, Conn) as we described previously [14]. Ang II solution was prepared by dissolving 13 mg Ang II in 2.21 mL PBS. SU6656 was prepared by dissolving 25 mg SU6656 in 3.52 mL DMSO, followed by dilution with 5.4 mL to final concentration.

### Western Blotting

Proteins were extracted from the isolated mesenteric vessel beds of mice or from the cultured human coronary artery SMCs (Lonza, Walkersville, MD) by using RIPA buffer (Cell Signaling Technology, Inc., Boston, MA) supplemented with complete protease inhibitor cocktail (Roche, Indianapolis, IN) and phosphatase inhibitors (Cell Signaling Technology, Inc., Boston, MA). Western blotting were performed as we described previously [15] by using antibodies to Src, phospho-Src (Tyr416), MLC, and phospho-MLC (Ser19) (Cell Signaling Technology, Inc., Boston, MA). Western blotting bands were scanned with HP Scanjet 7400c, and the band intensities were quantified with ImageJ software.

### Immunofluorescence Staining

Human coronary artery SMCs were seeded on cover slides, treated with SU6656 (5uM) or Vehicle for 30 min, then stimulated with Ang II (0.1nmol/L) for additional 10 min, followed by immunofluorescent staining and examination under a confocal microscope (Zeiss LSM 510 META) as we described previously [14].

### Statistics

All values are reported as mean ± SEM. Two-way ANOVA with repeated measures were used for comparisons of 2 factors at multiple levels. p<0.05 was considered statistically significant.

## Results

### Inhibition of SFK Lowers the BP Level in Ang II-Treated Mice

**Fig 1:**
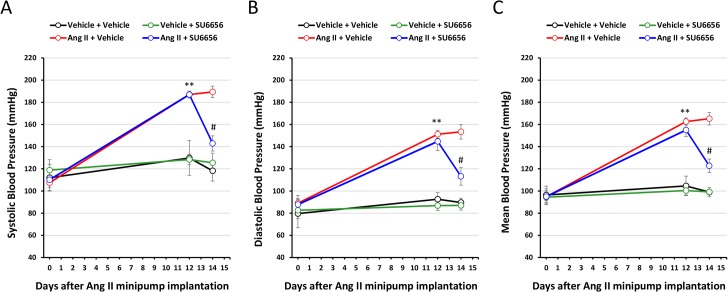
Inhibition of SFK lowers the BP level in Ang II-treated mice. Eight week-old male C57BL/6 mice were administered Ang II (1.4 mg/kg/d) or Vehicle via a subcutaneous minipump (14-day release), and starting from 13^th^ day, received daily i.p. injection of SU6656 (8 mg/kg/d) or Vehicle for 2 days. The systolic **(A)**, diastolic **(B)** and mean **(C)** BP were measured in mice by a tail-cuff method at day 0 (prior to Ang II treatment), day 12 (prior to SU6656 treatment), and day 14. n = 10 per group; **p<0.01, Ang II vs. Vehicle; #p<0.05, Ang II+SU6656 vs. Ang II.

To induce hypertension, we administered Ang II to mice via a subcutaneous minipump. Compared to control treatment (Vehicle), Ang II treatment markedly increased systolic (187 vs. 107 mmHg), diastolic (151 vs. 89 mmHg), and mean (165 vs. 95 mmHg) arterial BP, which reached plateau on day 12 (Fig 1). Injection of SFK inhibitor SU6656 significantly lowered the level of BP in Ang II-treated mice (systolic, 147 vs. 187 mmHg; diastolic, 113 vs. 145 mmHg; and mean, 123 vs. 155 mmHg) (Fig 1), however, did not altered the BP level in Vehicle-treated mice. These results suggest that SFK contribute to Ang II-induced hypertension.

### Inhibition of SFK Blunts Ang II-Induced MLC Phosphorylation in the Resistant Vessels

**Fig 2:**
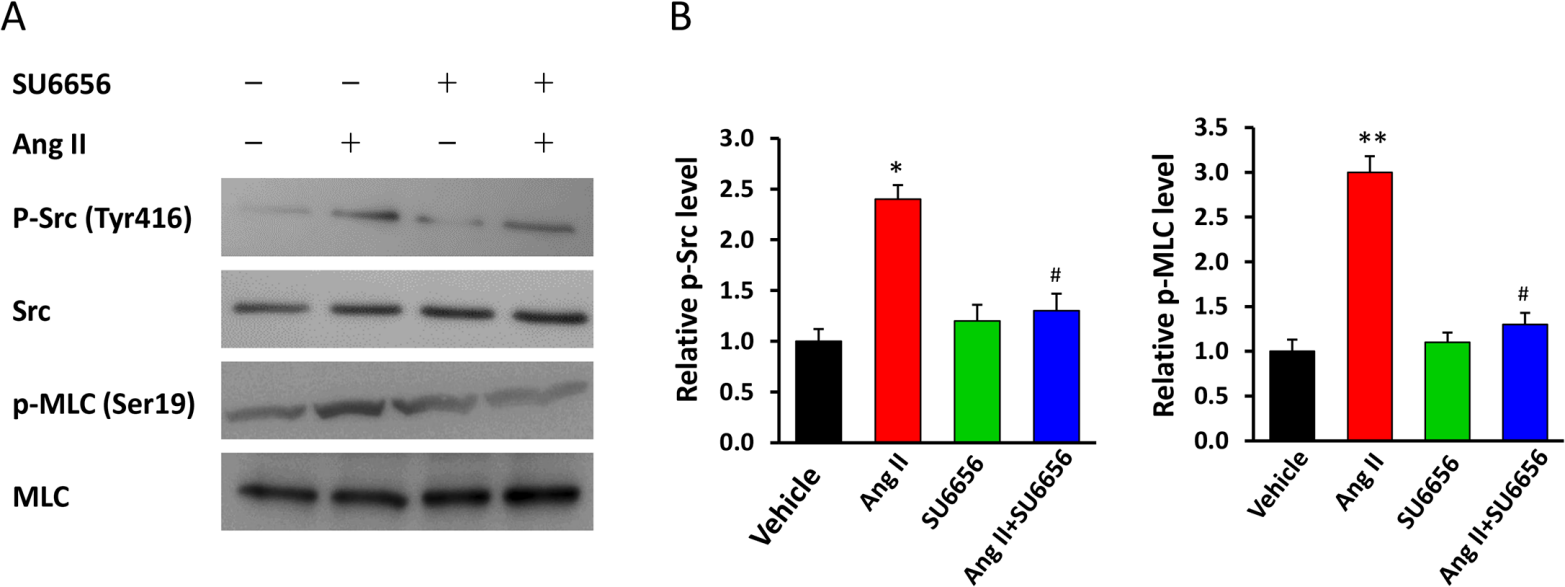
Inhibition of Src attenuates Ang II-induced MLC phosphorylation in mesenteric vessels. After last BP measurements (at day 14) in mice described in Fig 1, the mice were euthanized, and their mesenteric vessels were isolated and subjected to Western blotting analyses for the phosphorylated Src and MLC. (**A**) Representative and (**B**) quantification of Western blotting analyses in mice treated with Vehicle (–), Ang II, SU6656, or Ang II+SU6656. The intensities of the phospho-protein bands were quantified densitometrically, normalized to the total proteins, and expressed as fold differences relative to the values in the Vehicle group (“SU6656[–] Ang II [–])”. n = 5/group; *p<0.05, **p<0.01, Ang II vs. Vehicle; # p<0.05, Ang II+SU6656 vs. Ang II.

Because phosphorylation of the MLC is a key regulatory event that allows myosin to interact with actin and generate force, we analyzed the levels of phospho-MLC in the mesenteric arteries, which are typical resistant vessels. Ang II treatment markedly increased the levels of phospho-MLC as well as phospho-Src (Fig 2). However, SU6656 treatment diminished Ang II-induced increase in phospho-MLC (Fig 2). These results suggest that SFK may contribute to Ang II-induced BP elevation, at least partially, by augmenting MLC phosphorylation; and SFK inhibition attenuates Ang II-induced BP elevation by blunting MLC phosphorylation.

### SFK Contribute to the Ang II-Induced MLC Phosphorylation and Contractility in Human Arterial SMCs

**Fig 3:**
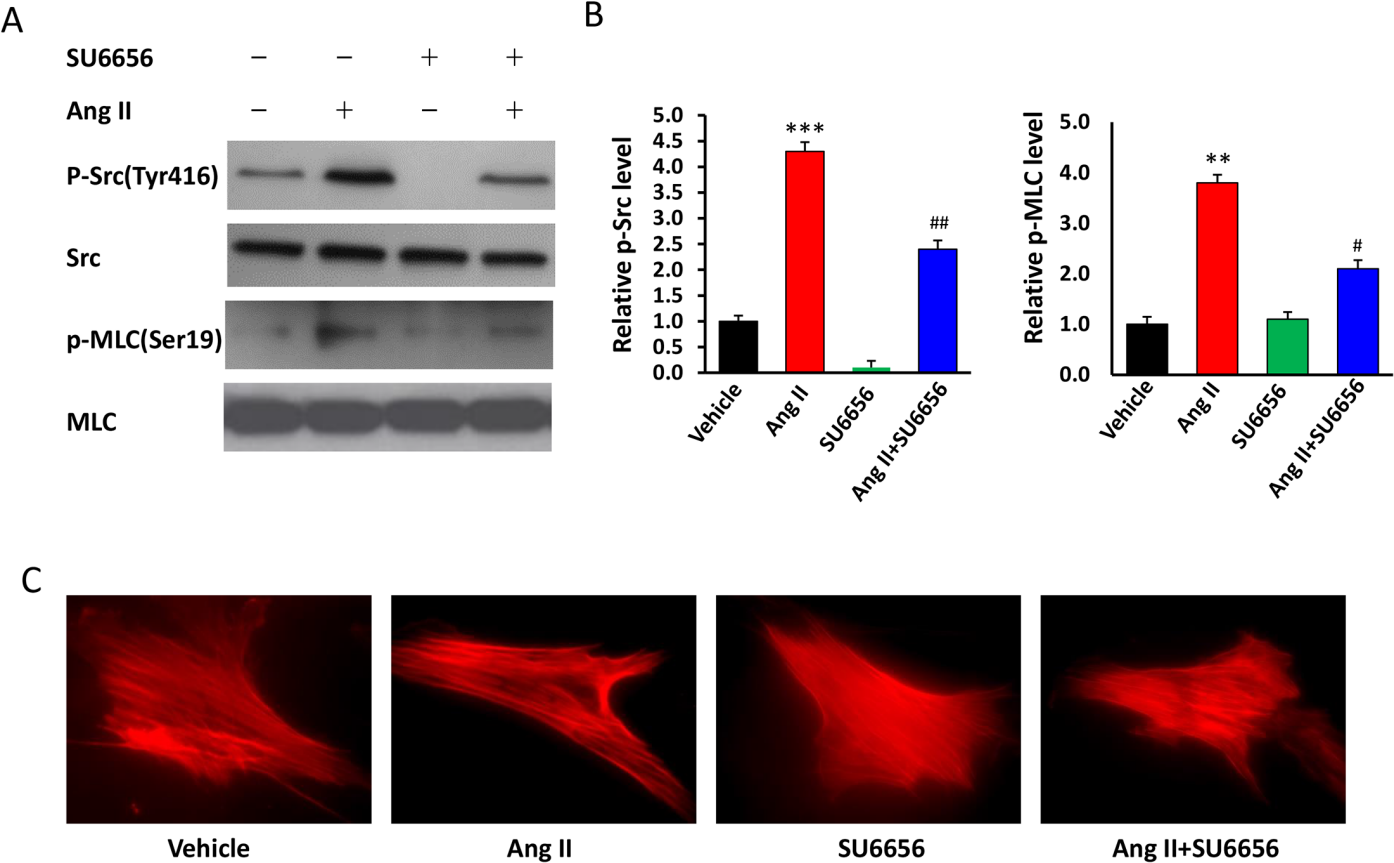
Src contributes to the Ang II-induced MLC phosphorylation and contractility in human VSMCs. The human coronary artery SMCs in culture were treated with SU6656 (5 uM) or Vehicle (–) for 30 min, then stimulated with Ang II (0.1 nmol/L) or Vehicle (–) for additional 10 min. (**A**) Representative and (**B**) quantification of Western blotting analyses of phosphorylated Src and MLC. Levels of the phospho-proteins were normalized to total proteins and expressed as fold differences relative to the values in the Vehicle group. n = 5/group, **p<0.01, ***p<0.001, Ang II vs. Vehicle; #p<0.05, ##p<0.01, Ang II+SU6656 vs. Ang II. (**C**) Immunofluorescent staining of Actin. Results are representative of 3 independent experiments.

Because the structure of smooth muscle myosin and the phosphorylation sites on MLC are conserved across species including mice and humans, we sought to determine whether the regulatory role of SFK in the vascular smooth muscle in mice also applies to humans. Human coronary artery SMCs in culture were treated with SU6656 for 30 min, then with Ang II for additional 10 min, and followed by Western blotting analyses of phosphorylated Src and MLC and by immunofluorescence staining of actin to indicate cell contractility. We found that Ang II treatment markedly increased Src and MLC phosphorylation and SMC contraction, and that these changes were abrogated by pretreatment with SU6656 (Fig 3). These results are consistent with our observations in mouse vessels and confirm that Ang II induced SMC contractile function is mediated by SFK.

## Discussion

In this study, our results demonstrate that the SFK play a crucial role in Ang II-induced hypertension. Inhibition of SFK lowers the level of systemic BP and smooth-muscle MLC phosphorylation in the resistant vessels of Ang II-treated mice and attenuates the induced contractility of cultured arterial SMCs from humans.

Although both vascular contraction and SFK activation are induced by Ang II, and the activation of SFK in vascular system has been implicated in various Ang II-related cellular processes (e.g., cell proliferation and migration), until now the extent to which SFK contribute to Ang II-mediated regulation of BP is unknown. Our results for the first time demonstrate that SFK directly regulate Ang II-induced vascular contraction and influence BP. Moreover, our results provide an additional mechanistic link between Ang II and vasoconstriction via SFK-enhanced MLC phosphorylation in SMCs. These findings suggest the exciting possibility that targeted inhibition of SFK may provide a new therapeutic opportunity in the treatment of hypertension.

Mice or humans express 9 closely related SFK, and they differ in their tissue expression patterns. Src, Fyn, and Yes are the major SFK in the vascular tissue [16–18]. SU6656 inhibits all SFK members [19], and the available antibodies are not specific for the phosphorylated forms of individual SFK, so we used antibodies against Src that cross-react with Fyn and Yes. Nevertheless, the results reported here cannot identify which specific types of SFK are activated by Ang II signaling. We have begun a series of experiments with mice deficient in one or more SFK to characterize the roles of the individual family members.

**Fig 4:**
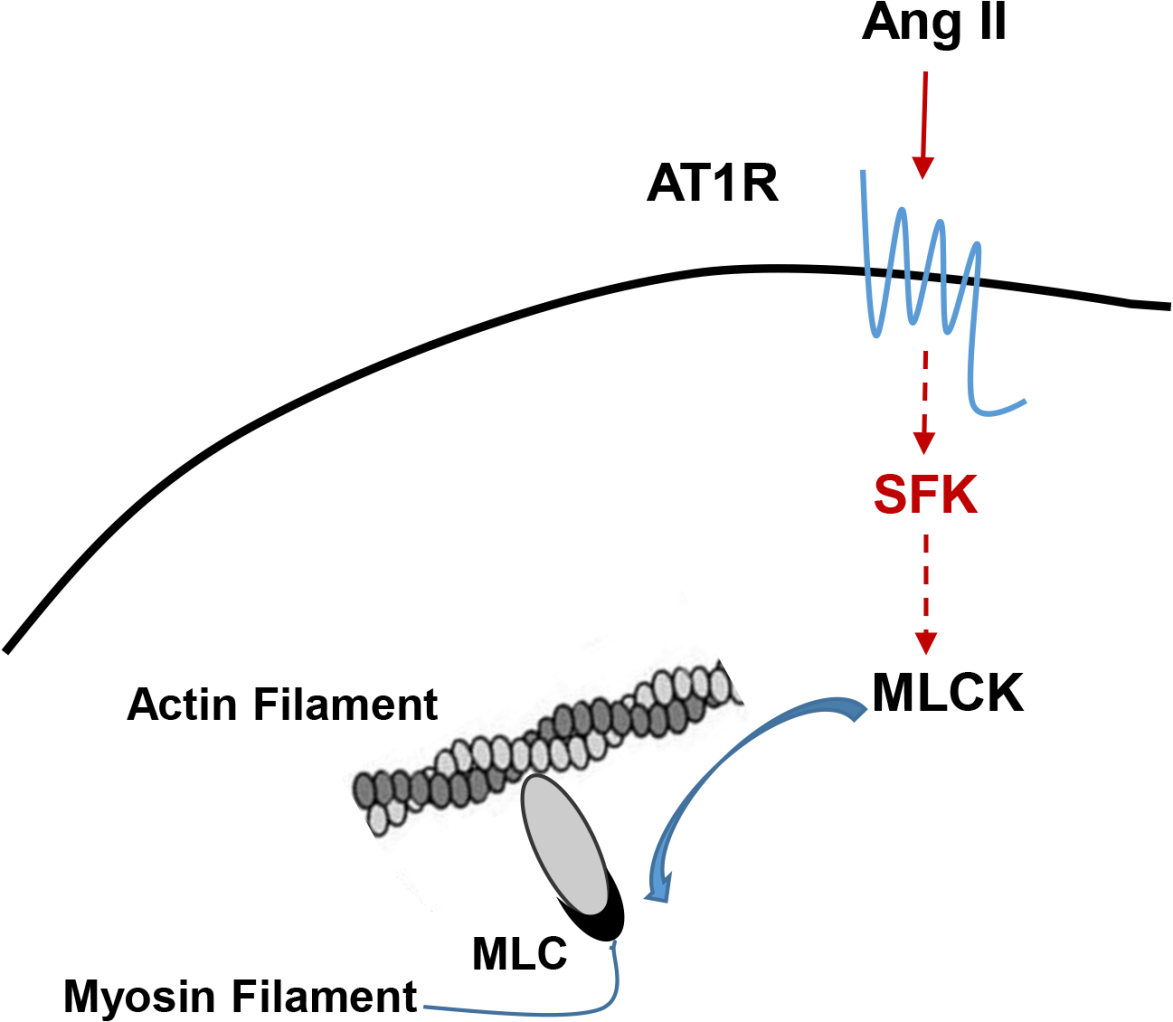
A novel mechanism by which Ang II induces smooth muscle contraction. Interactions between Ang II and AT1R triggers SFK phosphorylation, which in turn, via tyrosine kinase activity and currently-unknown enzymatic substrates, results in MLCK phosphorylation at Serine 19 and activation of smooth muscle contractile machinery.

Although our results clearly show that SFK enhance MLC phosphorylation, it is less likely that SFK directly phosphorylate the serine residues on MLC (Fig 4). Investigations on more detailed molecular mechanisms, such as whether SFK enhances the activity of Ca2+/calmodulin-dependent MLC kinase, are currently underway.

In conclusion, the results presented here demonstrate that SFK play a crucial role in Ang II mediated BP regulation by modulating the phosphorylation states of MLC in vascular SMCs. The mechanism identified by our studies may have the potential to be modulated for enhancing the effectiveness of hypertension therapy.

## References

[1] Go AS, Mozaffarian D, Roger VL, Benjamin EJ, Berry JD, Blaha MJ, et al Executive summary: heart disease and stroke statistics—2014 update: a report from the American Heart Association. Circulation. 2014;129(3):399–410. 10.1161/01.cir.0000442015.53336.12 . 24446411

[2] Sever PS, Messerli FH. Hypertension management 2011: optimal combination therapy2011 2011-10-01 00:00:00. 2499–506 p. 10.1093/eurheartj/ehr177 21697169

[3] Walsh MP. Vascular smooth muscle myosin light chain diphosphorylation: mechanism, function, and pathological implications. IUBMB Life. 2011;63(11):987–1000. 10.1002/iub.527 . 21990256

[4] Ikebe R, Reardon S, Mitsui T, Ikebe M. Role of the N-terminal Region of the Regulatory Light Chain in the Dephosphorylation of Myosin by Myosin Light Chain Phosphatase. Journal of Biological Chemistry. 1999;274(42):30122–6. 10.1074/jbc.274.42.30122 10514500

[5] Schiffrin EL, Park JB, Intengan HD, Touyz RM. Correction of Arterial Structure and Endothelial Dysfunction in Human Essential Hypertension by the Angiotensin Receptor Antagonist Losartan. Circulation. 2000;101(14):1653–9. 10.1161/01.cir.101.14.1653 10758046

[6] Garrido AM, Griendling KK. NADPH oxidases and angiotensin II receptor signaling. Molecular and Cellular Endocrinology. 2009;302(2):148–58. 10.1016/j.mce.2008.11.003. 10.1016/j.mce.2008.11.003 19059306 PMC2835147

[7] Nguyen Dinh Cat A, Touyz R. Cell Signaling of Angiotensin II on Vascular Tone: Novel Mechanisms. Current Hypertension Reports. 2011;13(2):122–8. 10.1007/s11906-011-0187-x 21274755

[8] Mehta PK, Griendling KK. Angiotensin II cell signaling: physiological and pathological effects in the cardiovascular system2007 2007-01-01 00:00:00. C82-C97 p. 10.1152/ajpcell.00287.2006 16870827

[9] Higuchi S, Ohtsu H, Suzuki H, Shirai H, Frank GD, Eguchi S. Angiotensin II signal transduction through the AT1 receptor: novel insights into mechanisms and pathophysiology. Clin Sci (Lond). 2007;112(8):417–28. 10.1042/CS20060342 . 17346243

[10] Tang DD, Anfinogenova Y. Physiologic Properties and Regulation of the Actin Cytoskeleton in Vascular Smooth Muscle. Journal of Cardiovascular Pharmacology and Therapeutics. 2008;13(2):130–40. 10.1177/1074248407313737 18212360 PMC2396785

[11] Abe K, Nakashima H, Ishida M, Miho N, Sawano M, Soe NN, et al Angiotensin II-Induced Osteopontin Expression in Vascular Smooth Muscle Cells Involves Gq/11, Ras, ERK, Src and Ets-1. Hypertens Res. 2008;31(5):987–98. 10.1291/hypres.31.987 18712054

[12] Mugabe BE, Yaghini FA, Song CY, Buharalioglu CK, Waters CM, Malik KU. Angiotensin II-Induced Migration of Vascular Smooth Muscle Cells Is Mediated by p38 Mitogen-Activated Protein Kinase-Activated c-Src through Spleen Tyrosine Kinase and Epidermal Growth Factor Receptor Transactivation. Journal of Pharmacology and Experimental Therapeutics. 2010;332(1):116–24. 10.1124/jpet.109.157552 19797620 PMC2802473

[13] Touyz RM1, Wu XH, He G, Park JB, Chen X, Vacher J, et al Role of c-Src in the regulation of vascular contraction and Ca2+ signaling by angiotensin II in human vascular smooth muscle cells. J Hypertens. 2001;19(3):441–9.(3):441–9. 11288814 10.1097/00004872-200103000-00012

[14] Zhou J, Zhu Y, Cheng M, Dinesh D, Thorne T, Poh KK, et al Regulation of Vascular Contractility and Blood Pressure by the E2F2 Transcription Factor. Circulation. 2009;120(13):1213–21. 10.1161/circulationaha.109.859207 19752322 PMC2785027

[15] Cheng M, Huang K, Zhou J, Yan D, Tang YL, Zhao TC, et al A critical role of Src family kinase in SDF-1/CXCR4-mediated bone-marrow progenitor cell recruitment to the ischemic heart. J Mol Cell Cardiol. 2015;81:49–53. 10.1016/j.yjmcc.2015.01.024 . 25655934 PMC4380859

[16] Oda Y, Renaux B, Bjorge J, Saifeddine M, Fujita DJ, Hollenberg MD. cSrc is a major cytosolic tyrosine kinase in vascular tissue. Canadian journal of physiology and pharmacology. 1999;77(8):606–17. . 10543724

[17] Corey SJ, Day RM. Shockingly: the loss of Lyn leads to leakiness. Blood. 2013;122(25):4009–10. 10.1182/blood-2013-10-533158 24335031 PMC3862277

[18] Eliceiri BP, Paul R, Schwartzberg PL, Hood JD, Leng J, Cheresh DA. Selective requirement for Src kinases during VEGF-induced angiogenesis and vascular permeability. Molecular cell. 1999;4(6):915–24. . 10635317 10.1016/s1097-2765(00)80221-x

[19] Tatton L, Morley GM, Chopra R, Khwaja A. The Src-selective kinase inhibitor PP1 also inhibits Kit and Bcr-Abl tyrosine kinases. The Journal of biological chemistry. 2003;278(7):4847–53. 10.1074/jbc.M209321200 . 12475982

